# Collagen Triple Helix Repeat Containing 1 (CTHRC1) acts via ERK-dependent induction of MMP9 to promote invasion of colorectal cancer cells

**DOI:** 10.18632/oncotarget.1714

**Published:** 2014-01-18

**Authors:** Hee Cheol Kim, Yong Sung Kim, Hyun-Woo Oh, Kwoneel Kim, Sang-Seok Oh, Jong-Tae Kim, Bo Yeon Kim, Seon-Jin Lee, Yong-Kyung Choe, Dong Hyeok Kim, Seok-Hyung Kim, Seoung Wan Chae, Kwang Dong Kim, Hee Gu Lee

**Affiliations:** ^1^ Biomedical Genomics Research Center, Korea Research Institute of Bioscience and Biotechnology, Daejeon, South Korea; ^2^ Division of Applied Life Science, PMBBRC, Gyeongsang National University, Jinju, South Korea; ^3^ Department of Surgery, Samsung Medical Center, Sungkyunkwan University School of Medicine, Seoul, South Korea; ^4^ Department of Pathology, Samsung Medical Center, Sungkyunkwan University School of Medicine, Seoul, South Korea; ^5^ Department of Pathology, Kangbuk Samsung Hospital, Sungkyunkwan University School of Medicine, Seoul, South Korea

**Keywords:** CTHRC1, ERK, MMP9, Invasion, Colorectal cancer

## Abstract

Collagen triple helix repeat-containing 1 (CTHRC1) is known to be aberrantly upregulated in most human solid tumors, although the functional roles of CTHRC1 in colorectal cancer remain unclear. In this study, we investigated the occurrence of CTHRC1 upregulation and its role in vivo and in vitro. The expression profile and clinical importance of CTHRC1 were examined by reverse transcription-polymerase chain reaction and immunohistochemical analyses in normal and tumor patient samples. CTHRC1 was detectable in normal tissues, but also was highly expressed in tumor specimens. CTHRC1 upregulation was significantly associated with demethylation of the CTHRC1 promoter in colon cancer cell lines and tumor tissues. Clinicopathologic analyses showed that nodal status and expression of CTHRC1 (95% CI 0.999–3.984, p=0.05) were significant prognostic factors for disease-free survival. Promoter CpG methylation and hypermethylation status were measured by bisulfite sequencing and pyrosequencing analysis. Furthermore, we showed that overexpression of CTHRC1 in the SW480 and HT-29 cell lines increased invasiveness, an effect mediated by extracellular signal-regulated kinase (ERK)-dependent upregulation of matrix metalloproteinase 9 (MMP9). Consistent with this, we found that knockdown of CTHRC1 attenuated ERK activation and cancer cell invasivity. These results demonstrate that CTHRC1 expression is elevated in human colon cancer cell lines and clinical specimens, and promotes cancer cell invasivity through ERK-dependent induction of MMP9 expression. Our results further suggest that high levels of CTHRC1 expression are associated with poor clinical outcomes.

## INTRODUCTION

Colon cancer is a major health problem worldwide, and among colon cancer patients with a clinical onset greater than 55 years of age, mortality rates of have actually increased. Genetic and epigenetic changes in the genome have been investigated for their potential contribution to the transformation of normal cells into cancer cells [1–3]. The role of DNA methylation in particular has been considered an important mechanism of tumorigenicity [4–7]. Changes in the cellular characteristics of transformed cells, such as uncontrolled proliferation and metastatic potential, are the major cause of mortality in tumor patients. Metastasis is associated with changes in cell adherence, levels of metalloprotease expression, and cellular mobility [8, 9].

Collagen triple helix repeat-containing 1 (CTHRC1) was identified as a novel secreted protein in injured and diseased arteries that inhibits collagen expression and promotes cell migration [10]. In addition to functioning in the context of arterial injury, CTHRC1 has been reported to act as a positive regulator of osteoblastic bone formation to increase bone mass [11]. In this latter report, Kimura et al. suggested an anabolic approach for the treatment of osteoporosis based on the use of CTHRC1-null and transgenic mice. It was recently reported that CTHRC1 selectively activates the planar cell polarity (PCP) pathway of Wnt, but inhibits the canonical Wnt pathway [12]. Although CTHRC1 expression has been observed in human solid cancers [13, 14], and aberrant expression of CTHRC1 is associated with cancer tissue invasion and metastasis in melanoma [13], how CTHRC1 induces these phenotypes has remained unknown.

Matrix metalloproteinases (MMPs), which are important in cell growth and proliferation, also play a key role in cancer cell migration [15, 16], with considerable evidence implicating MMPs in the degradation of the extracellular matrix (ECM) during the metastatic process. The ECM plays an important role in the modulation of MMPs in physiological and pathological states [17, 18]. The degree of overexpression of some MMPs has been reported to correlate with the stage of disease and/or prognosis [19]. MMP9 degrades type I collagen under certain conditions, allowing tumor cells to break from the site of the primary tumor, leading to invasion and metastasis [20].

In this study, we identified CTHRC1 as a colorectal cancer-related gene whose expression is regulated by an epigenetic mechanism, namely DNA methylation. In addition to nodal status, CTHRC1 expression was found to be a significant prognostic factor for disease-free survival. CTHRCl-overexpressing colon cancer cell showed greater invasive capacity and higher levels of MMP9 expression than did control (mock-transfected) cells. Upregulation of MMP9 was dependent on activation of extracellular signal-regulated kinase (ERK) in CTHRCl-overexpressing cells. Taken together, these results indicate that oncologic outcomes are worse in patients whose tumors express high levels of CTHRC1, which promotes invasivity of tumor cell *via* ERK-dependent induction of MMP9 expression.

## RESULTS

### Identification of CTHRC1 as a colorectal cancer-associated gene

**Figure 1:**
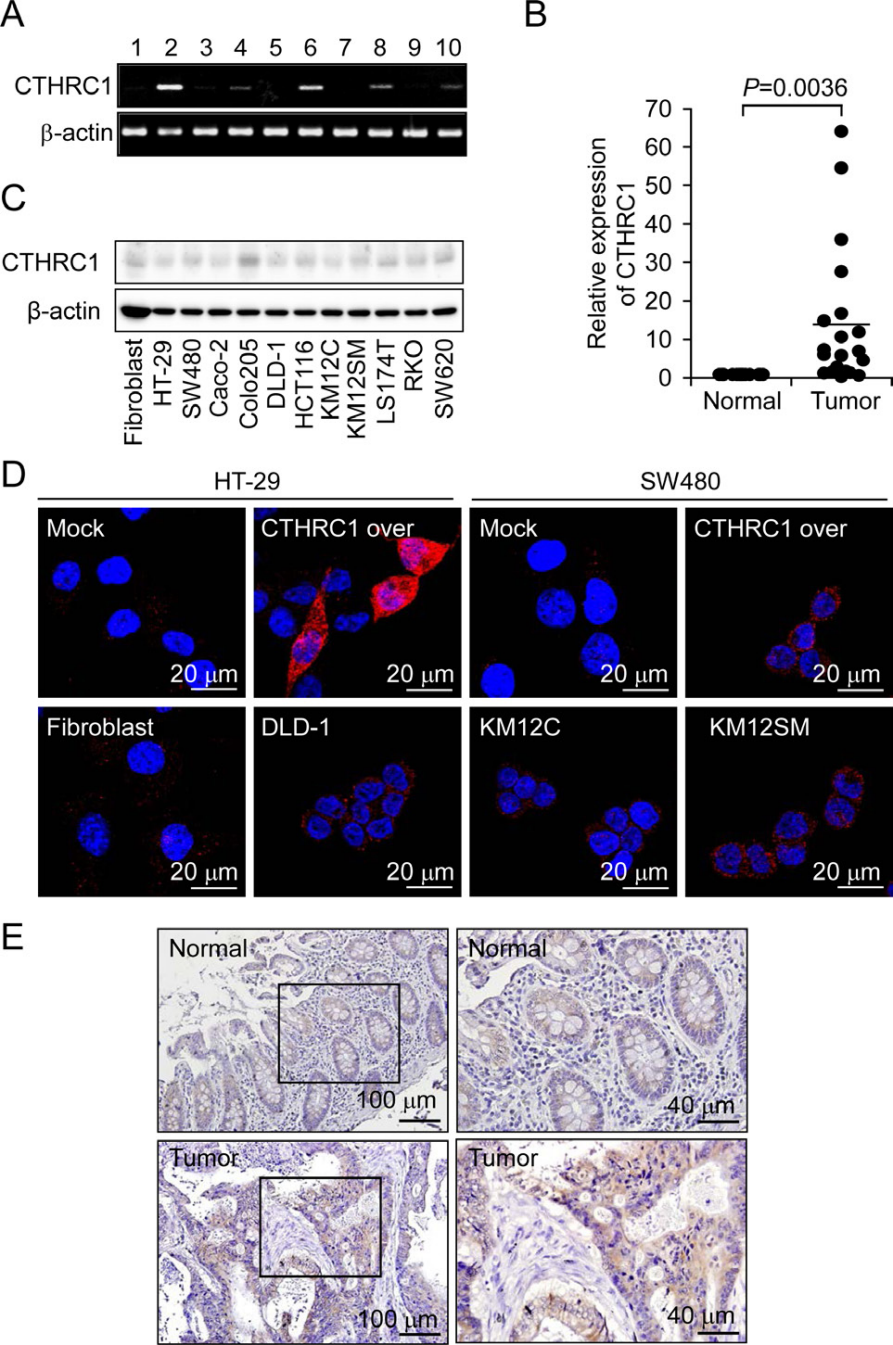
Upregulation of CTHRC1 mRNA expression in colon cancer Cancer tissues were 1ysed and analyzed by RT-PCR (A) and quantitative RT-PCR (B). The β-actin gene was used as an internal control. The basal levels of CTHRC1 were detected in Primary fibroblast and various colorectal cancer cell lines by Western blot analysis (C) and immunocytochemistry (D). (E) Representative immunohistochemical staining of CTHRC1 is shown in normal tissues and tumor tissues.

To explore differentially expressed genes between normal tissue and colorectal cancer tissue, we performed a microarray analysis on 66 tumor samples and 9 normal samples using a 48K Illumina oligonucleotide chip (Illumina Inc.), identifying *CTHRC1* as a gene upregulated in colorectal cancer as described previously [21]. A comparison of *CTHRC1* transcript levels in colon cancer tissues and normal tissue confirmed these results (Fig. 1A and B). We also examined the basal expression level of CTHRC1 in primary fibroblast and human colorectal cancer cell lines such as HT-29, SW480, DLD-1, KM12C, and KM12SM by Western blot analysis and laser confocal microscope (Fig. 1C and D). We also used immunohistochemistry to investigate the possibility that CTHRC1 protein might be a prognostic marker CTHRC1 was detected slight expression levels in normal mucosal epithelial cells and colorectal cancer lesions (Fig. 1E). In those results, CTHRC1 could be expressed in normal cells and tissues, but also high expression in tumor cells and tissues. These data suggest that CTHRC1 is upregulated in colorectal cancer and, as such, may be a colorectal cancer-associated gene.

### Epigenetic regulation of CTHRC1 gene expression in colorectal cancer

**Figure 2:**
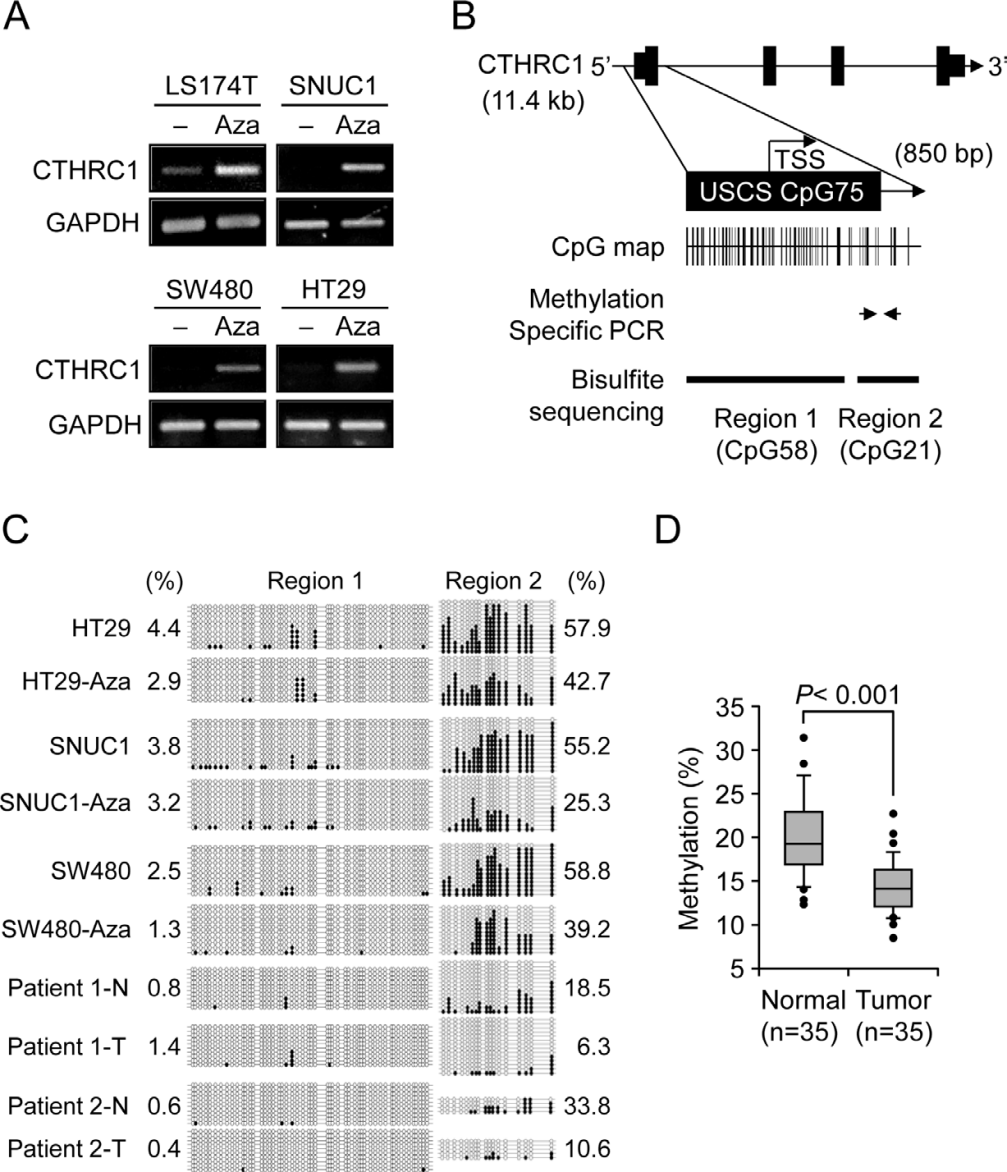
Correlation of CTHRC1 expression with CpG methylation in the prompter region (A) Effects of 5-Aza-dC on CTHRC1 expression. CTHRC1 mRNA was detected by RT-PCR in LS174T, SNU-C1, SW480, HT29 cells treated with 10 μM 5-Aza-dC. Each value is the mean ± SD of three independent experiments. GAPDH was used as an internal control. (B) Schematic representation of the structure of the *CTHRC1* gene on human chromosome 8q22.3. CpG islands were predicted using the University of California, Santa Cruz, genome browser (http://genome.ucsc.edu/). Two regions were selected for bisulfite sequencing analysis; three CpG sites in Region 2 subjected to pyrosequencing analysis are indicated. (C) Bisulfite sequencing analysis of the *CTHRC1* promoter in three colon cancer cell lines and two pairs of normal (N) and tumorous (T) colon tissues. Open circles, unmethylated CpG sites; filled circles, methylated CpG sites. Each row represents the results for a single clone. Numerical values for Region 1 (left) and Region 2 (right) represent the mean percentages of CpG sites that were methylated for each cell line or tissue. (D) Pyrosequencing analysis of three CpG sites in CTHRC1 (see Fig. 2B) from 35 paired primary colon tumor tissues and adjacent normal tissues. CTHRC1 methylation was sharply decreased in tumors compared with normal tissues (p < 0.001). The box plot shows the median, and 25^th^ and 75^th^ percentiles; dots represent outliers

To investigate whether *CTHRC1* gene expression is regulated by an epigenetic mechanism, specifically promoter CpG methylation, we treated colon cancer cell lines that showed low CTHRC1 expression (LS174T, SNUC1, SW480, and HT-29) with the demethylating agent, 5-Aza-dC, and then examined CTHRC1 mRNA expression by RT-PCR. This analysis showed that CTHRC1 expression was restored or greatly increased in 5-Aza-dC-treated cells compared to controls (Fig. 2A), suggesting that *CTHRC1* gene expression is regulated by promoter CpG methylation. We also performed a bisulfite sequencing analysis of CpG islands in the promoter region of *CTHRC1* to determine which CpG region is critically associated with restoration of CTHRC1 expression after 5-Aza-dC treatment. A very low level of methylation was observed in CpG sites from Region 1 (Fig. 2B), and 5-Aza-dC treatment had no effect on methylation in this region in any of the colon cancer cell lines tested (Fig. 2C, left panel). In contrast, all colon cancer cell lines showed CpG hypermethylation (57%, on average) in Region 2 before 5-Aza-dC treatment (Fig. 2C, right panels). CpG methylation levels were decreased in all cell lines after 5-Aza-dC treatment: from 57.9% to 42.7% in HT29 cells, from 55.2% to 25.3% in SNUC1 cells, and from 58.8% to 39.2% in SW480 cells (Fig. 2C, right panels). These results suggest that *CTHRC1* gene expression may be regulated by CpG methylation in the exon 1 region rather than in the 5′-upstream region.

### Association of CTHRC1 promoter demethylation with CTHRC1 upregulation in primary colorectal cancer tissue

Figure 2C shows that, similar to the results in colon cancer cell lines, CpG methylation levels in Region 1 were also very low in primary colorectal cancer compared to paired normal tissues. Specifically, in normal samples, CpG in Region 2 was hypermethylated and the methylation status was greatly decreased in the associated tumor tissue, from 18.5% to 6.3% in Patient 1, and from 33.8% to 10.6% in Patient 2 (Fig. 2C, right panels). These results suggest that the exon 1 region of *CTHRC1* may be critically associated with regulation of *CTHRC1* gene expression in primary colorectal tumors as well as in colon cancer cell lines. For a more quantitative estimation of methylation status using pyrosequencing analysis, we selected five CpG sites in Region 2 that showed a great change in CpG methylation in cell lines after 5-Aza-dC treatment or in primary tumors compared to normal tissues. The analysis was then performed on 35 paired clinical samples to evaluate the clinical implications of demethylation. As shown in Figure 2D, the mean methylation status in five CpG sites was significantly lower in tumor tissues (14.30%) ± 3.01%) than in normal tissues (19.88% ± 4.52%; p < 0.001). These results suggest that hypermethylation at these five CpG sites may be an intrinsic characteristic of normal colon tissue, whereas demethylation of these CpG sites frequently occurred in colon tumor tissues, giving rise to *CTHRC1* upregulation.

### Relationship between CTHRC1/MMP9 expressions and clinicopathologic characteristics and oncologic outcomes

**Figure 3:**
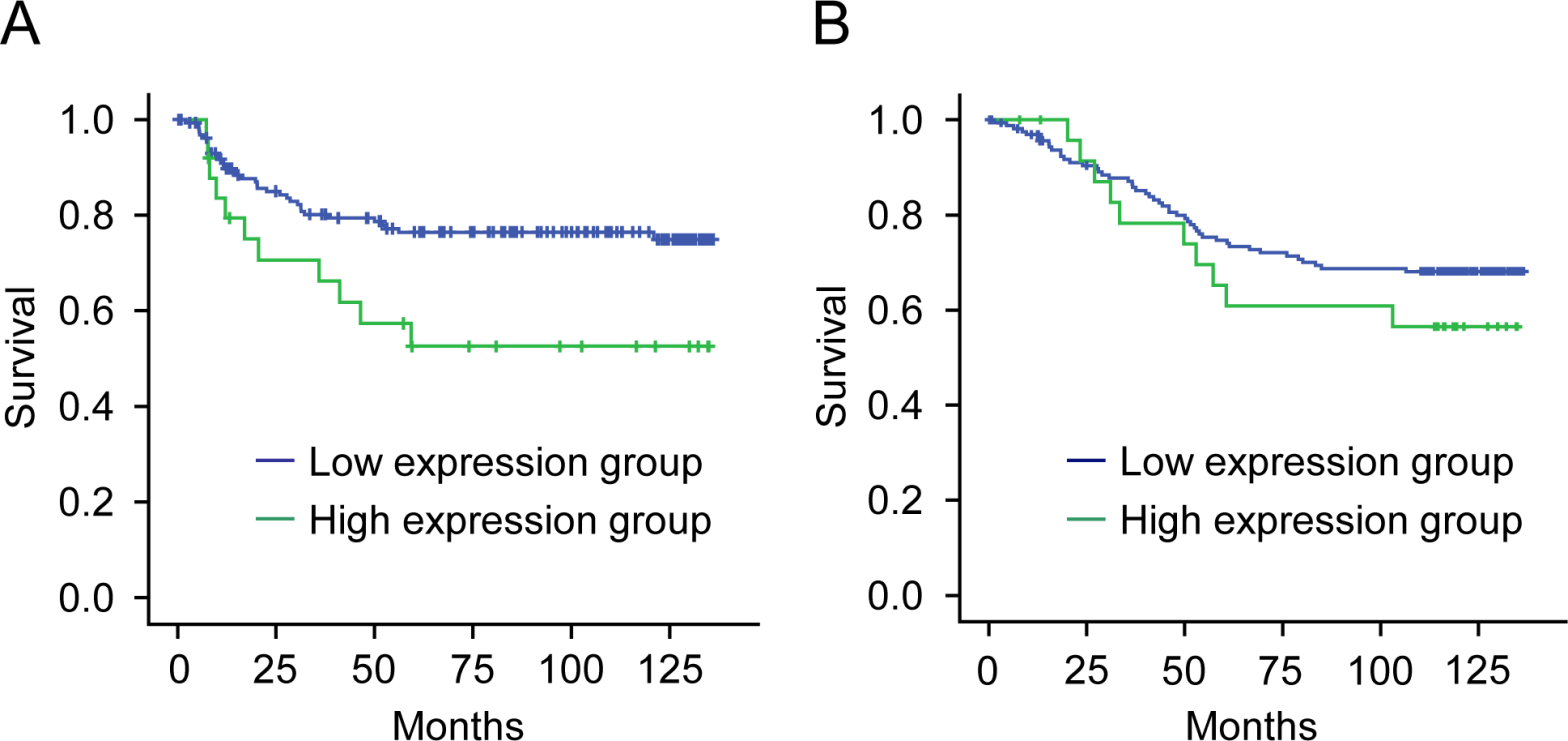
Survival of patients in relation to CTHRC1 expression in colorectal cancers (A) Disease-free survival as a function of the expression of CTHRC1 in colorectal cancers (p = 0.021). (B) Overall survival as a function of the expression of CTHRC1 in colorectal cancers (p = 0.306).

Mean value of immune-expression of CTHRC1 was 3.27±1.68 (SD) and median value was 4 (range, 0–8). Thirteen patients (6.9%) did not express CTHRC1, at all. Twenty-five patients (13.3%) were classified a high-expressing and 163 patients (86.7%) were categorized as low-expressing with respect to CTHRC1. There were no differences in clinicopathologic factors between the two groups (Supplementary Table 1). As shown in Figure 3A, 5-year disease-free survival rates were higher in the low-expression group (77.2%) than in the high-expression group (57.1%; p = 0.021). Overall 5-year survival rates trended higher in the low-expression group (74.7%) than in the high-expression group (60.5%), but this difference did not reach statistical significance (p = 0.306; Fig. 3B). A multivariate analysis showed that nodal status and CTHRC1 expression were significant prognostic factors for disease-free survival (95% CI 0.999-3.984, p = 0.05; Table 1). Nodal status was also a significant prognostic factor for overall survival (Supplementary Table 2).

**Table 1:** Cox expression analysis (disease free survival)

Variables	SE	*p*-value	RR	95% Cl
Sex	0.305	0.419	1.28	0.704–2.326
Age	0.308	0.619	0.858	0.469–1.569
Location	0.151	0.644	1.072	0.797–1.443
Cell type	0.458	0.198	1.803	0.735–4.422
T stage	0.458	0.802	1.122	0.457–2.753
N stage	0.34	0.001	3.238	1.664–6.301
CTHRC1	0.353	0.05	1.995	0.999–3.984
MMP9	0.343	0.922	1.034	0.528–2.026

Abbreviation: SE, standard error; RR, relative ratio; 95% CI, 95% confidence interval

*Determined by Cox's proportion hazard model

Regarding MMP9 expression in colorectal cancer tissues, mean value of immune-expression was 1.39±1.77 (SD) and median value was 1 (range, 0–8). Eighty nine (47.3%) did not express MMP9. 48 patients (25.5%) were classified a high-expressing and 140 patients (74.5%) were categorized as low-expressing. MMP9 expression was related to vascular invasion in tumor tissue (p=0.044) but did not show the any association with other clinicopathologic factors analyzed. MMP9 did not have the significant impact in disease-free survival either overall survival rate. The expressions of MMP9 was weakly correlated to the expressions if CTHRC1 in colorectal cancer tissue (R=0.171, p=0.019).

### Contribution of CTHRC1 to the invasiveness of colon cancer cells

**Figure 4:**
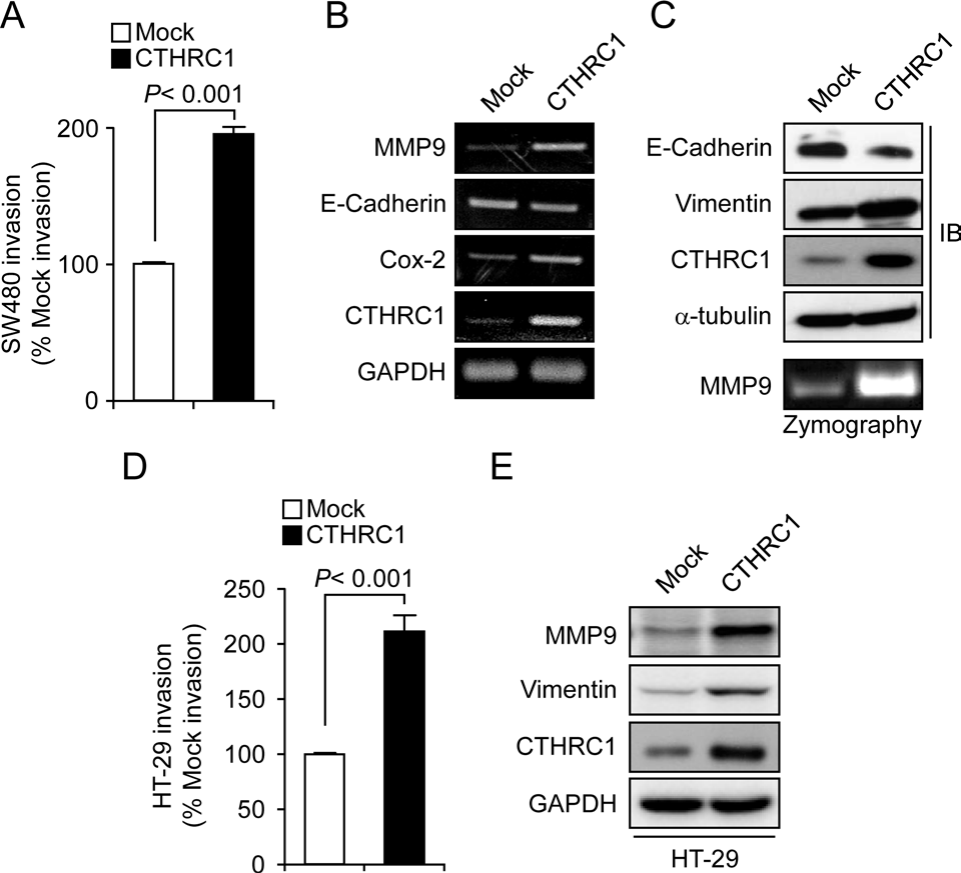
CTHRC-1 induces invasion *via* upregulation of MMP9 (A) The invasive behavior of SW480-Mock and SW480-CTHRC1 cells was assessed in 24-well, modified Boyden chambers. Results are expressed as means ± SEMs from three experiments (p < 0.001, compared to control cells). mRNA (B) and protein (C) levels of EMT markers in SW480-Mock and SW480-CTHRC1 cells were measured by RT-PCR and Western blotting, respectively. SW480-Mock and SW480-CTHRC1 (C) and HT-29-Mock and HT-29-CTHRC1 (D) cells were cultured in serum-free medium for 72 hours. Conditioned medium was collected and concentrated by centrifugation Samples containing equal amounts of protein were analyzed by zymogram gel assays. The gelatinolytic activity of MMP9 was detected by Coomassie blue staining as clear bands on the gel. (E) The levels of vimentin and MMP-9 protein in HT-29-Mock and HT-29-CTHRC1 cells were measured by Western blotting

To determine the function of CTHRC1 as a putative oncogene in colon cancer cells, we examined cell proliferation, β-catenin activity, and invasiveness in a CTHRCl-overexpressing SW480 cell line (SW480-CTHRC1). The invasiveness of SW480-CTHRC1 and HT-29 cells was increased almost 2-fold compared with that of vector-transfected (SW480-Mock and HT-29-Mock) control cells (Fig. 4A and D), whereas proliferative activity and spontaneous β-catenin activity were not significantly different (Supplementary Fig. 1). To identify genes regulated under CTHRC1-overexpressed conditions, we examined the expression of epithelial-mesenchymal transition (EMT)-related genes using RT-PCR. MMP9 and COX2 mRNA were increased in SW480-CTHRC1 cells, and E-cadherin was slightly decreased (Fig. 4B). Immunoblotting confirmed that the EMT-inhibitory protein, E-cadherin, was decreased, whereas the EMT-associated proteins, vimentin and MMP9, were increased (Fig. 4C and E). MMP9 expression was especially sensitive to CTHRC1 overexpression at both mRNA and protein levels. Consistent with the observed changes in MMP9 expression, gelatin zymography showed that MMP9 activity was also increased under CTHRCl-overexpressed conditions (Fig. 4C). To confirm the relationship between CTHRC1 and MMP9, we performed conventional RT-PCR and real-time RT-PCR on RNA originating from the same patients. Although MMP9 was detected in some CTHRC1-negative patients, CTHRC1 was expressed in most colon cancer patients, and CTHRC1-positive tissues expressed MMP9 (Supplementary Fig. 2A and B). These results suggest that CTHRC1 positively regulates MMP9 expression to induce tumor invasion.

### CTHRC1 upregulates MMP9 via ERK activation

**Figure 5:**
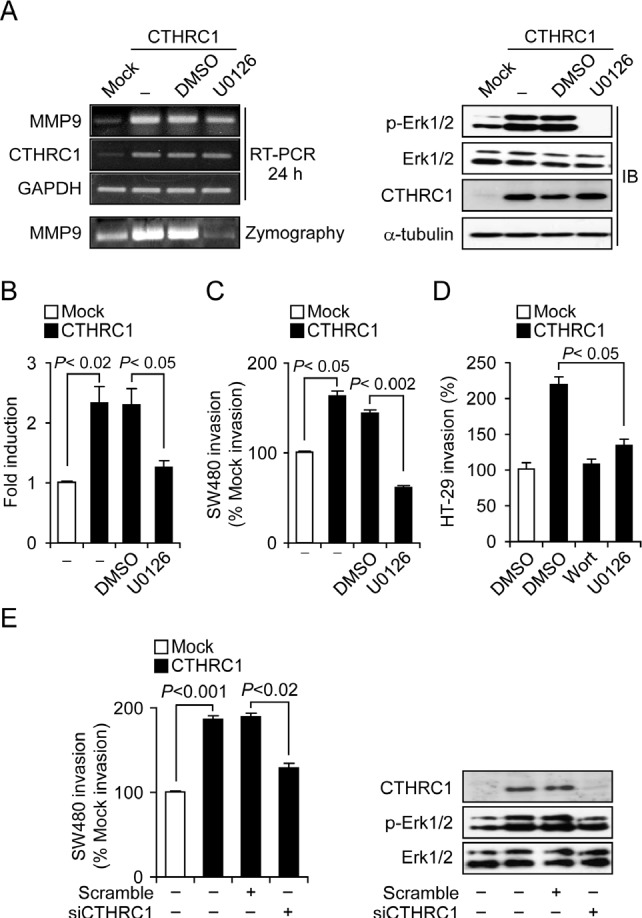
CTHRC1 promotes invasion of SW480 cells through activation of the MEK-ERK signaling pathway (A) Effects of U0126 on CTHRC1 overexpression-induced increases in phospho-ERK levels and MMP9 expression. SW480-CTHRC1 cells were treated with DMSO or 10 μM U0126 for 24 hours. (B) Luciferase reporter assay. Transcriptional activation of the ELK1 promoter by increased ERK signaling was assessed in SW480 cells using Dual-Luciferase Assays; relative response ratios are reported. Cells were cotransfected with the ELK1 reporter vector and pRL-TK vector, used as an internal control reporter vector. Each bar represents mean ± SEM (p < 0.02, p < 0.05). Cell invasion by SW480-CTHRC1 cells (C) and HT-29-CTHRC1 (D) cells was assessed in the presence of the ERK inhibitor U0126 (10 μM for 24 hours) and wortmannin (25 nM for 24 hours). (E) The invasive ability of SW480-Mock and SW480-CTHRC1 cells and that of their corresponding stable transfectants with siRNA-mediated knockdown of CTHRC1 was analyzed using invasion assays.

To determine how CTHRC1 regulates MMP9 expression, we treated SW480-CTHRC1 cells with inhibitors of phospholipase C, MEK1/2, c-Jun N-terminal kinase (JNK), p38 mitogen-activated protein kinase (MAPK), NF-KB, or Racl. The MEK1/2 inhibitor, U0126, specifically inhibited MMP9 expression in SW480-CTHRC1 cells (Supplementary Fig. 3). Notably, the levels of activated ERK were higher in SW480-CTHRC1 cells than in SW480-Mock cells, and U0126 effectively decreased both ERK mRNA and protein (Fig. 5A). It would reduce the levels of phosphorylated ERK without affecting ERK mRNA or protein. To confirm activation of the ERK pathway, we performed luciferase reporter assays using an ERK-responsive ELK1 promoter. ELK1-luciferase activity was higher in SW480-CTHRC1 cells, and this activity disappeared following treatment with the MEK inhibitor, U0126 (Fig. 5B). Moreover, the invasiveness of SW480-CTHRC1 and HT-29-CTHRC1 cells was decreased by ERK inhibition (Fig. 5C and D), and both the increased invasiveness and ERK activation in these cells were diminished by CTHRC1 knockdown (Fig. 5E). Collectively, these results suggest that CTHRC1 acts through ERK-mediated upregulation of MMP9 to promote increased invasiveness of colon cancer cells.

## DISCUSSION

In the present study, we demonstrated using microarray analysis that CTHRC1 is a colorectal cancer-related protein that is differentially expressed between human normal and tumor patient tissues. Recently, it was reported that some human solid tumors, including metastatic melanoma and invasive breast cancer, showed higher expression of CTHRC1 [13, 14]. However, the regulation and function of CTHRC1 in colorectal cancer and its relationship with tumor progression have not been reported. In this study, we first described the aberrant elevated expression of CTHRC1 in colon cancer, which suggests that CTHRC1, which is slightly expressed in normal mucosal epithelial tissues, may be a novel oncogene (Fig. 1). CTHRC1 in injured tissue has been shown to lead to a reduction in collagen type I, which might play a role in vascular remodeling *via* matrix deposition and cell migration [10]. The elevated levels of CTHRC1 in colorectal cancer patients could suggest an important role of this protein in tumor progression.

Tumor progression is a multistep process resulting from altered gene expression through genetic and epigenetic modulation [22]. Epigenetic modulation is known to be involved in the transformation from normal cell to cancer cell. Hypomethylation of DNA, in particular, has been proposed to induce tumorigenesis by several mechanisms [5, 23]. Hypomethylation of human growth hormone, y-globin, and HRAS in cancer tissues compared with their normal counterparts was reported in early genome-wide and gene-specific hypomethylation studies [24, 25]. The promoter region *of CTHRC1* was also shown to be hypermethylated in some cell lines and normal tissue, and it was reported that *CTHRC1* expression is induced by inhibition of DNA methyltransferase. In the current study, we showed for the first time that CTHRC1 is an epigenetic target in colorectal cancer and that the frequent upregulation of CTHRC1 observed in colon cancer may be due to a CpG demethylation event in the exon 1 region of the gene.

To minimize bias in our analysis of the association of CTHRC1 protein expression with clinicopathologic features, we used only tumor tissues for which complete clinicopathologic and long-term follow-up data were available from patients who had been operated on in the same year. Because there was no concrete criterion for CTHRC1 protein expression, tumors were assigned to low and high CTHRC1-expressing groups using cutoff values of 4 and 5. These cutoffs were set based on a consideration of median and average CTHRC1 expression in tumor tissues, respectively. Clinical results were similar using either value. Clinicopathologic variables, including stage, were comparable in low- and high-expression groups. However, recurrence rates were significantly different between low (22%) and high (44%) CTHRC1-expressing groups (p = 0.026), and were even more dramatically different at the extreme ends of the two groups (15% vs. 54%). Disease-free survival in the high-expression group was also poorer. Based on this clinical data, CTHRC1 could be considered an effective and novel prognostic marker for the prediction of recurrence or metastasis. Patients that express high levels of CTHRC1 might need aggressive chemotherapy and surveillance. Notably, patients in this group could be good candidates for biologic agents that affect the ERK pathway. Verification of tumor responses to biologic agents according to the expression of CTHRC1 in vitro and vivo will be the next research step in the development of new treatment strategies based on such agents.

MMPs are associated with cancer-cell invasion and metastasis. Activation of MMPs has been detected in almost all types of human cancer and is closely correlated with advanced tumor stage, increased invasion and metastasis, and shortened survival time. Since CTHRC1 is expressed in several cancer models [13, 14] and has recently been implicated in vascular diseases [10], we hypothesized that CTHRC1 might regulate colorectal cancer tissue invasion and metastasis through modulation of MMP9. MMP9 level is a valuable indicator of colorectal cancer patients who are at high risk of developing cancer recurrence [26], and increase of MMP9 expression has been shown to promote invasion and metastasis through activation of focal adhesion kinase/extracellular signal-regulated kinase/matrix metalloproteinase-9 axis in human hepatocellular carcinoma [27]. In present study, MMP9 expression in colorectal cancer tissue with variable cutoff criteria of immune-reactivity did not show the association with clinical features such as cell type, stage, and oncologic outcomes of survivals except vascular invasion. Although it is not enough to say that MMP9 is bad prognostic factor, there are still possibility for MMP9 to be associated with tumor invasion because we found the relationship between MMP9 and vascular invasion. The expressions between CTHRC1 and MMP9 in colorectal cancer tissues revealed the weak correlations. With this data of immune-expression in colorectal cancer tissues, it is very difficult to speculate the close mechanism between CTHRC1 and MMP9. However, considering the diverse networks of signaling in tissue level and our experimental results with cancer cells, we could guess the relationship between two proteins. CTHRC1 gain-of-function and the resulting increased invasion of colon cancer cell lines, demonstrated here, suggest that CTHRC1 has a crucial role in tumor invasion and metastasis. Consistent with this, siRNA-mediated CTHRC1 knockdown significantly reduced the invasivity of colon cancer cells. Therefore, another mechanism by which CTHRC1 promotes tumor invasion may be related to its role in reducing the cleavage of collagen by ECM-digesting components, thus making it easier for tumor cells to migrate into the adjacent tissues. The increased protease activity may also facilitate intravasation and extravasation processes.

Previous studies have described several alternative functions of CTHRC1 in addition to signal transduction in tumor and vascular cells. Although CTHRC1 inhibits TGF-β signaling, which induces phosphorylation of Smad2/3 in smooth muscle cells [28, 29] and collagen type I expression in keloid fibroblasts [30], we could not confirm such an inhibitory effect in SW480-CTHRC1 cells (data not shown). It may be that the function of CTHRC1 in SW480 cells does not involve TGF-β-mediated signaling. Cell-surface-anchored CTHRC1 binds to Wnt, Fzd and Ror2 proteins, and is involved in selective activation of the PCP pathway, which inhibits the Wnt canonical pathway [12]. The PCP pathway can induce mobility [30] and promote metastasis in several tumor models, including melanoma, gastric cancer and breast cancer, by activating Rac and JNK [31–33]. In the current study, SW480-CTHRC1 and HT-29-CTHRC1 cells showed greater invasive behavior than SW480-Mock and HT-29-CTHRC1 control cells. Overexpression of CTHRC1 significantly enhanced MMP9 expression, an effect that was dependent on ERK activation and MMP9 induction by CTHRC1. This action did not involve Rac1 activation or the JNK pathway because neither the Rac1 inhibitor NSC23766 nor the JNK inhibitor SP600125 inhibited MMP9 expression in SW480-CTHRC1 cells. From these results, we infer that the PCP pathway is not likely associated with the enhanced invasive capacity of SW480-CTHRC1 cells.

Activated ERK levels were higher in SW480-CTHRC1 cells than in SW480-Mock control cells, and the MEK1/2 inhibitor U0126 specifically blocked MMP9 expression at both transcriptional and protein levels. The MEK/ERK pathway controls MMP9 expression in various cells [34–37], and MMP9 contributes to the invasion, migration, and metastasis of cancer cells [38–41]. In the current study, the invasiveness of SW480-CTHRC1 cells was diminished by treatment with the MEK1/2 inhibitor, and both ERK activation and invasivity were decreased by knockdown of CTHRC1. Although the question of how CTHRC1 activates the ERK pathway remains, induction of MMP9 via the ERK pathway is suggested as the major mechanism by which CTHRC1 enhances migration and invasiveness.

In conclusion, we demonstrated that CTHRC1 is highly expressed in colorectal cancer and its expression is correlated with unfavorable clinicopathologic features and methylation status. We propose that CTHRC1 promotes cancer cell invasiveness through activation of ERK and subsequent induction of MMP9 expression. Moreover, we suggest that high expression of this protein may be a useful prognostic marker in colorectal cancer. Because CTHRC1 is a secreted protein, developing a neutralizing antibody that suppresses its protease activity should be highly feasible. Insight into the pro-metastatic role of CTHRC1 may open a new avenue for the development of cancer therapies designed to inhibit metastasis.

## MATERIALS AND METHODS

### Ethics statement

This study was approved by the Institutional Review Board of Samsung Medical Center (Seoul, Korea).

### Patients and tissue microarray construction

A total of 188 patients with colorectal adenocarcinoma who underwent surgical resection for a primary colorectal cancer at Samsung Medical Center in 2000 and were consecutively identified based on a prospectively maintained colorectal cancer database, were enrolled in this study. The exclusion criteria were non-curative resection, cancer recurrence, double primary cancer, familial adenomatous polyposis (FAP) or hereditary nonpolyposis colorectal cancer (HNPCC), patients whose paraffin blocks were not available, uninformative cases of immunohistochemical staining, and cases lost within 1 month after operation. The sex ratio was 114:74, and the mean age was 57.4 years (range, 28–84; SD, 11.8). Median follow-up duration was 116 months (range, 2-136). Tumor areas used for tissue microarrays were selected by a single pathologist (SHK). A manual arraying device (MTA-1; Beecher Instruments Inc., Sun Prairie, WI, USA) was used for extraction of five 1.0-mm cores from each case: two from the invasive tumor and one from normal mucosa.

### Cell culture and generation of stable transfectants overexpressing CTHRC1

All human colon cancer cell lines were maintained in Dulbecco's modified Eagle's medium (DMEM; Sigma) supplemented with 10% (v/v) heat-inactivated fetal bovine serum (FBS; Sigma) and 1% penicillin/streptomycin (Lonza), and were cultured at 37°C in a humidified chamber with 5% CO_2_. Human CTHRC1 cDNA was amplified by polymerase chain reaction (PCR) using the following pair of primers: 5′-gat atc atg cga ccc cag ggc-3′ (forward) and 5′-etc gag tta atg gtg atg gtg atg atg ttt tgg tag ttc ttc aat aat gat gc-3′. The PCR product was cloned into the His-tagging expression vector, pcDNA3.1/hygro(+) (Invitrogen). SW480 and HT-29 cells were transfected with the CTHRC1 expression plasmid using the Lipofectamine 2000 transfection reagent (Invitrogen), following the instructions of the manufacturer. After 24 hours of incubation, cells were selected by culturing in the presence of 0.2 mg/ml hygromycin B (Calbiochem).

### Immunohistochemistry and pathologic evaluations

Immunohistochemistry was performed on 3-mm tissue microarray sections of normal and tumor tissues (4-(μm thick), as described previously [42], using an anti-CTHRC1 antibody (diluted 1:100; Abeam (ab85739)). Slides were evaluated for CTHRC1 staining by two pathologists (SHK & SWC). If there was a discrepancy in individual scores, both pathologists re-evaluated the slides together to reach a consensus before combining the individual scores. The annotation process included an estimation of the intensity of immunoreactivity for CTHRC1 and MMP9 (negative, 0; weak, 1; moderate, 2; strong, 3) and fraction (%) of CTHRC1 and MMP9-positive cells (<5%, 0; 5–25%, 1; 25–50%, 2; 50–75%, 3; > 75%>, 4). The total score was calculated as intensity × fraction. All tumors were categorized as high or low CTHRC1-expressing based on a cutoff score of 5 in CTHRC1 and on cutoff score of 3 in MMP9.

### Bisulfite sequencing

Genomic DNA (1 μg) from cancer cells or clinical samples was modified by sodium bisulfite using the EZ DNA Methylation kit (ZYMO Research) according to the manufacturer's instructions. The bisulfite sequencing analysis was performed at two regions of CpG islands in the CTHRC1 promoter; Region 1 contains 51 CpG sites within the 5′-upstream region of the CpG island, and Region 2 contains 17 CpG sites included in the 3′-downstream region of the CpG island (Fig. 2B). The following MSP primer sets were designed to amplify these two regions using the MethPrimer program (http://www.urogene.org/methprimer/index.html): Region 1, 5′-GTG TTT TTA AAA TGT TTA TTT AGG-3′ (forward) and 5′-AAC AAA AAC AAC AAA AAA CC-3′ (reverse), yielding a 475-bp product; and Region 2, 5′-TTT AAG GGG AAG TAAAAG G-3′ (forward) and 5′-AAA TTC CAA AAC TCA CTA CAC-3 (reverse), yielding a 254-bp product. Bisulfite-modified DNA was amplified by PCR in a 20-μ1 reaction containing each primer set using the following thermocycling conditions: 95°C for 1 minute, then 40 cycles of 95°C for 45 seconds, 51°C for 45 seconds and 72°C for 1 minute, followed by 72°C for 5 minutes. PCR products were cloned into the pGEM-T Easy Vector (Promega), and ten clones were randomly chosen for DNA sequencing.

### Pyrosequencing

Colon cancer cell lines showing low CTHRC1 expression were selected and seeded in 35-mm dishes at a density of 1 × 10^5^ cells/dish 1 day before drug treatment. Cells were treated with 10 μM 5-Aza-dC (Sigma) every 24 hours for 3 days, after which cells were lysed and DNA was prepared. Primers for pyrosequencing were designed with the PSQ Assay Design program (Biotage). Bisulfite-modified DNA (100 ng) was amplified by PCR in a 20-μl reaction containing a biotinylated forward primer (biotin-5′-GGG TGT TTG TTT GTA TAG TTG TG-3′) and a reverse primer (5′-CAA ATC CTT CTT CAC CTA TAA AT-3′) designed to yield products. Amplification reactions were performed using the follow conditions: 95°C for 5 minutes, then 35 cycles of 95°C for 30 seconds, 56°C for 30 seconds and 72°C for 30 seconds, followed by 72°C for 7 minutes. Pyrosequencing was performed using a sequencing primer (5′-TAA AAA ACA TAA CAC CCC-3′) on a PSQ HS 96A System (Biotage).

### Reverse transcription-polymerase chain reaction

Total RNA was prepared from cultured cells using the total RNA isolation solution, RiboEx (GeneAll, Seoul, Korea) according to the manufacturer's protocol. Reverse transcription-polymerase chain reaction (RT-PCR) was performed using a Verso 1-Step RT-PCR kit (Thermo Scientific, Abgene). The following primers were used for amplification: CTHRC1, 5′-TGG ACA CCC AAC TAC AAG CA-3′ and 5′ -GAA CAA GTG CCA ACC CAG AT-3′; MMP9, 5′-GAT GCG TGG AGA GTC AAA T-3′ and 5′-CAC CAA ACT GGA TGA CGA TG-3′; E-cadherin, 5′-TGA TTC TGC TGC TCT TGC TG-3′ and 5′-CGA GTC CCC TAG TCG TCC T-3′; Cox2, 5′-TTC AAA TGA GAT TGT GGA AAA ATT GCT-3′ and 5′-AGA TCA TCT CTG CCT GAG TAT CTT-3′; and glyceraldehyde 3-phosphate dehydrogenase (GAPDH), 5′-CCA TCA CCA TCT TCC AGG AG-3′ and 5′-ACA GTC TTC TGG GTG GCA GT-3′.

### Western blotting

Cells were lysed in a protein-extraction solution (PRO-PREP; iNtRON, Seongnam, Korea) on ice. Proteins in cell lysates were separated by sodium dodecyl sulfate-polyacrylamide gel electrophoresis (SDS-PAGE) on 12% gels and transferred to a polyvinylidene difluoride membrane (Millipore). Membranes were incubated with primary antibodies, as indicated, and then with horseradish peroxidase (HRP)-conjugated secondary antibodies (Santa Cruz Biotechnology). Immunoreactive bands were visualized using a chemiluminescent substrate (Millipore).

### Small interfering RNA (siRNA) experiments

CTHRC1 expression was knocked down with small interfering RNA (siRNA) using the synthetic duplex oligomers, 5′ -CCC AUU GAA GCU AUA AUU U-3′ and 5′-AAA UUA UAG CUU CAA UGG G-3′ (siCTHRCl), designed and purchased from Genolution Pharmaceuticals, Inc. (Seoul, Korea). siRNA oligoduplexes were transiently transfected using G-fectin Genolution Pharmaceuticals, Inc.) according to the manufacturer's protocol. After incubation for 48 hours, cells were lysed and the efficiency of siRNA-mediated target knockdown was confirmed by Western blotting.

### Cell invasion and luciferase reporter assay

*In vitro* cell invasion assays were performed using a modified Boyden chamber assay. Cells were added to the upper chamber of QCMTM 24-Well Cell Invasion Assay Kit (Millipore) inserts. Medium containing serum (10%) was utilized as the chemoattractant in the lower chamber. After 24 hours of incubation, cells that had invaded to the lower surface of the collagen-coated membrane were analyzed according to the manufacturer's instructions.

Cell extracts were prepared 24 hours after transfection, and luciferase activity was measured using the Dual-Luciferase Reporter Assay System (Promega). All experiments were performed three times in triplicate.

### Gelatin zymography

Cells were plated at a density of 6 × 10^6^ cells in 10-cm culture plates. After 16 hours, cells were washed with phosphate-buffered saline (PBS) and cultured in serum-free medium for 72 hours. Conditioned medium was collected and concentrated by centrifugation. Equal amounts of protein were mixed with non-reducing Laemmli sample buffer and resolved by SDS-PAGE on 10% gels containing 0.1% (w/v) gelatin. After incubation, MMP9 activity was detected by Coomassie blue staining as clear bands on the gel.

### Statistical analysis

Chi-square or Fisher's exact tests were used to assess the univariate associations of baseline characteristics. Survival was assessed using the Kaplan-Meier method. Cox proportional hazard models were used to assess the effects of various co-variables on survival. Correlation of expressions in tissues between CTHRC1 and MMP9 was analyzed using spearman test. All analyses were performed using the SPSS program (Ver. 19; IBM).

## Supplementary Figures and Tables




