# Supplementary material for: Collagen Triple Helix Repeat Containing 1 (CTHRC1) acts via ERK-dependent induction of MMP9 to promote invasion of colorectal cancer cells
Source: Oncotarget. 2014 Jan 18;5(2):519–29. doi: 10.18632/oncotarget.1714 (PMC3964226; doi:10.18632/oncotarget.1714)
Supplement: Supplementary file 1 [file oncotarget-05-519-s001.pdf]

Collagen Triple Helix Repeat Containing 1 (CTHRC1) acts via ERK-dependent induction of MMP9 to promote invasion of colorectal canc er cells – Kim et al

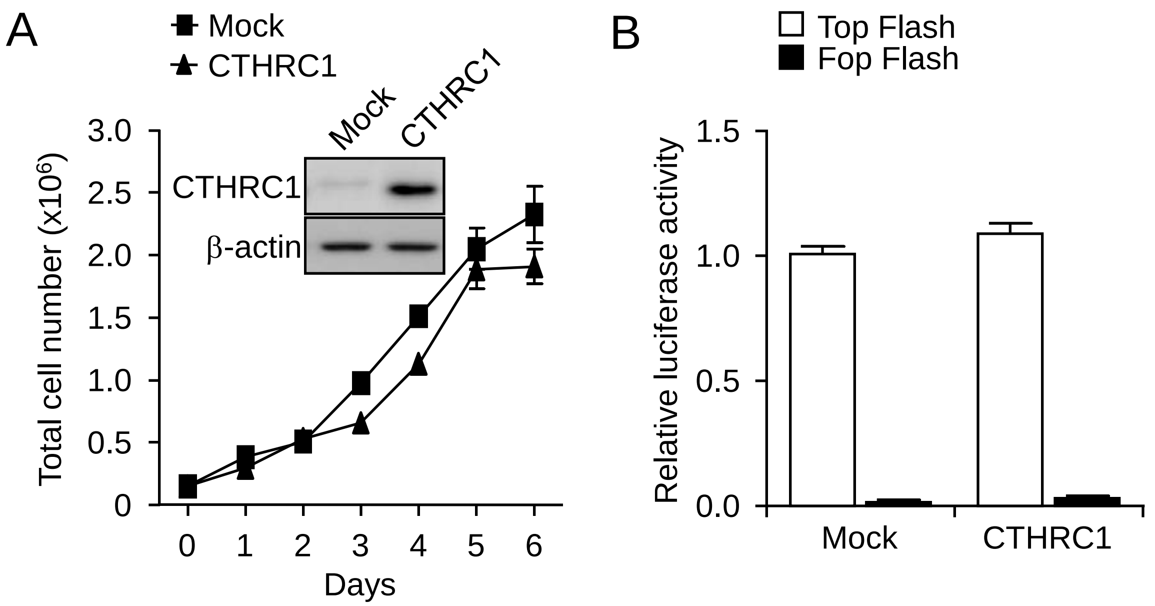

Supplement figure 1: (Left panel) Growth curves of transfected cells *in vitro*. Cell s were plated at a low density (5 × 10^4 per well), and cell numbers were counted every day. (Right panel) A control vector or a CTHRC1 overexpresed SW480 cell s are active β-catenin was coexpressed with the TCF/LEF reporter Top-Flash or a control reporter Fop-Flash in SW480 cells and luciferase activity measured after 24 h.

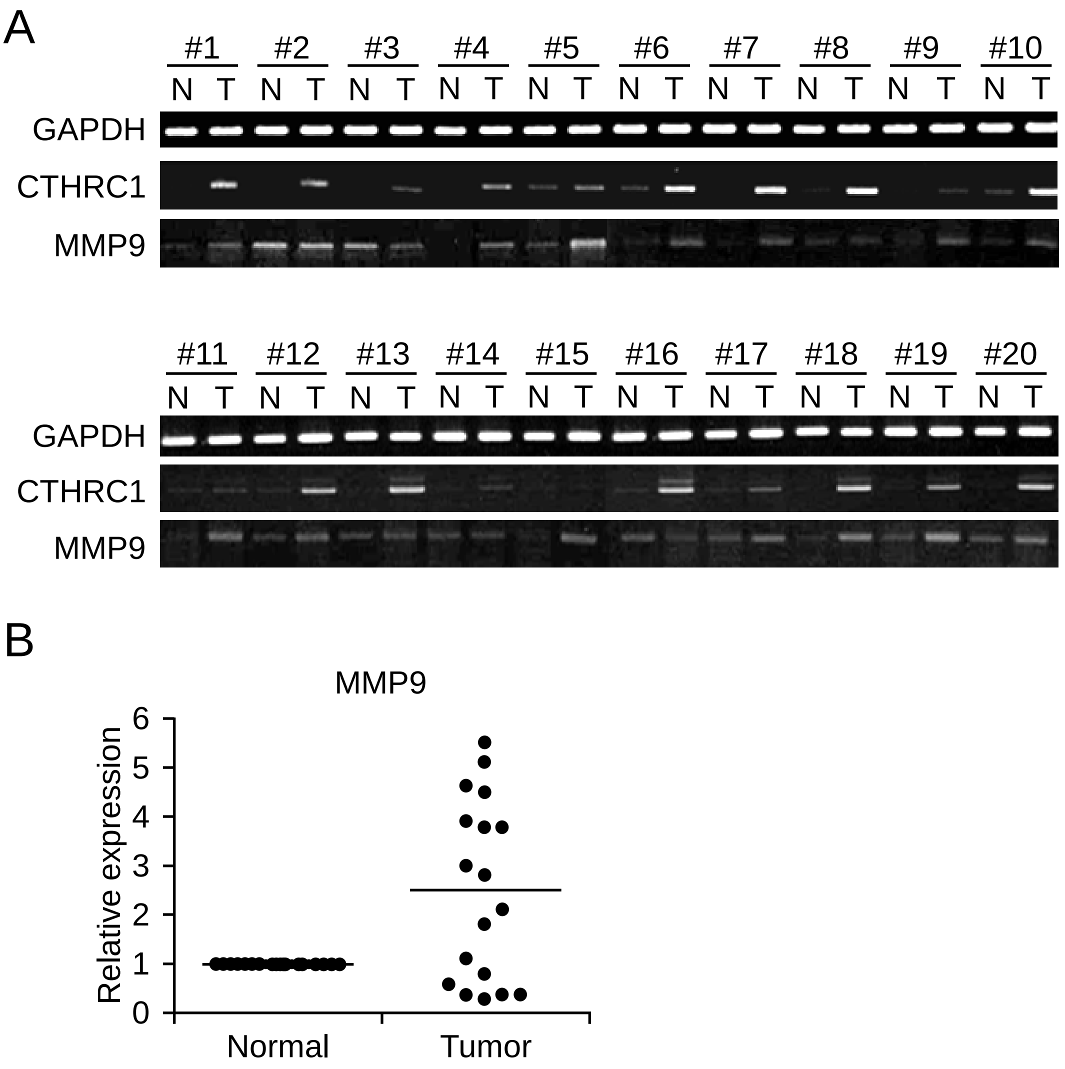

Supplement figure 2: Correlation between CTHRC1 and MMP9 in tumor tissue. To test whether CTHRC1 and MMP9 expression are correlated in cancer tissues, the transcripts from colon cancer tissues were analyzed using RT-PCR (A) and quantitative PCR (B) in terms of MMP9 expression.

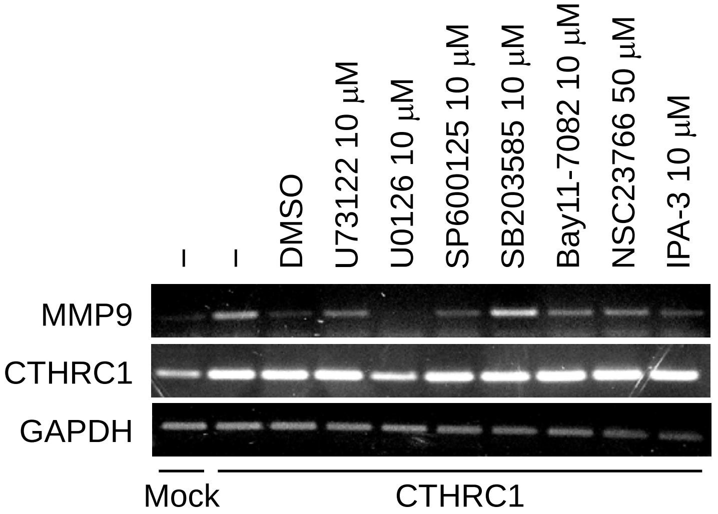

Supplement Figure 3. Rac1 is not involved in CTHRC1-mediated induct ion of MMP9. SW480-CTHRC1 cells were treated with various inhibito r for 24 hr. The level of MMP9 was examined by RT-PCR. U73122; pho spholipase C inhibitor, U0126; MEK inhibitor, SP600125; JNK inhibitor , SB203585; p38 kinase inhibitor, Bay11-7082; IKK inhibitor, NSC2376 6; Rac1 inhibitor, IPA-3; p21-activated kinase inhibitor III.
