# Supplementary material for: Collagen Triple Helix Repeat Containing 1 (CTHRC1) acts via ERK-dependent induction of MMP9 to promote invasion of colorectal cancer cells
Source: Oncotarget. 2014 Jan 18;5(2):519–29. doi: 10.18632/oncotarget.1714 (PMC3964226; doi:10.18632/oncotarget.1714)
Supplement: Supplementary file 2 [file oncotarget-05-519-s002.pdf]

Supplementary Table 1. Clinicopathologic features according to expression of CTHRC1

	Total	CTHRC1 expression		p-value
		Low/Negative	High	
Sex				0.944
Male	114	99	15	
Female	74	64	10	
Age				0.989
60>	98	85	13	
60≤	90	78	12	
Location				0.229
Colon	107	90	17	
Rectum	81	73	8	
Cell type				0.159
High grade	11	8	3	
Low grade	177	155	22	
T stage				0.219
T1/T2	31	29	2	
T3/T4	157	134	23	
N stage				0.5
N0	102	90	12	
N1,2	86	73	13	
Stage				0.478
I	23	22	1	
II	79	68	11	
III	83	70	13	
IV	3	3	0	
MMP9				0.084
Low/Negative	140	125	15	
High	48	38	10	

*Determined by chi-squre test or fisher's exact test

†High grade includes poorly differentiated, mucinous, and signet ring cell carcinoma; Low grade includes well and moderately differentiated adenocarcinoma

Supplementary Table 2. Cox regression analysis (overall survival)

Variables	SE	*p*-value	RR	95% CI
Sex	0.272	0.764	1.085	0.637 – 1.850
Age	0.272	0.179	1.441	0.846 – 2.455
Location	0.133	0.317	1.142	0.880 – 1.482
Cell type	0.461	0.177	1.863	0.755 – 4.594
T stage	0.442	0.189	1.787	0.751 – 4.251
N stage	0.28	0.007	2.121	1.226 – 3.670
CTHRC1	0.359	0.572	1.225	0.606 – 2.473

Abbreviation: SE, standard error; RR, relative ratio; 95% CI, 95% confidence interval

*Determined by Cox's proportion hazard model
